# The impact of vaccination on patients with COVID-19 during the wave of Omicron in Shanghai

**DOI:** 10.3389/fpubh.2022.1054313

**Published:** 2022-11-09

**Authors:** Chen Yu, Zhu Fengzhao, Wu Hongmei, Lei Zeyuan, Liu Yu, Guo Yuhang, Shen Rufei, Jia Qingzhu, Sun Xiaorong, Wang Xia, Song Caiping, Xu Zhi, Luo Chunmei

**Affiliations:** ^1^Department of Orthopaedics, Xinqiao Hospital, The Army Medical University, Chongqing, China; ^2^Department of Respiratory, Xinqiao Hospital, The Army Medical University, Chongqing, China; ^3^Department of Plastic Surgery, Xinqiao Hospital, The Army Medical University, Chongqing, China; ^4^Department of Endocrinology, Xinqiao Hospital, The Army Medical University, Chongqing, China; ^5^Department of Oncology, Xinqiao Hospital, The Army Medical University, Chongqing, China; ^6^Department of Office of the Hospital, Xinqiao Hospital, The Army Medical University, Chongqing, China

**Keywords:** COVID-19, Omicron, vaccination, asymptomatic, potential factors

## Abstract

**Background:**

The global health has been affected by the COVID-19 pandemic persistently, of which Omicron is currently the predominant variant. However, the impact of vaccination on Omicron remained uncertain.

**Objective:**

This study sought to explore the effect of vaccination on patients infected with Omicron.

**Methods:**

A retrospective observational cohort was conducted in the largest Fangcang shelter hospital in Shanghai from April 1 to May 30, 2022. The demographics, length of hospital stay, clinical symptoms, the comorbidities and vaccination status were recorded. Clinical outcomes of the vaccinated and non-vaccinated groups were compared and analyzed.

**Results:**

Of the 3,119 patients who fulfilled the eligibility criteria and were enrolled in the study, 2,226 (71.4%) patients had received nCoV-19 vaccine while 893 (28.6%) patients had not received it before admission. Patients in the vaccinated group had significantly shorter length of hospital stay than those in the unvaccinated group (15.48 ± 2.708 vs. 15.85 ± 3.102, *p* < 0.001). More asymptomatic patients were observed in the vaccinated group than the non-vaccinated (70.4 vs. 64.5%, *p* < 0.001). Further subgroup analysis demonstrated that the older the age, the more significant the difference was (*p* < 0.005).

**Conclusions:**

Vaccination was associated with a significant reduction in the severity of Omicron infection compared with no vaccination. Vaccination appears to make Omicron-infected people with milder symptoms than unvaccinated people. This suggests the potential effectiveness of current vaccines against Omicron.

## Introduction

The new coronavirus has posed a huge threat to global health since 2019. By 2 September 2022, 601,189,435 confirmed cases of COVID-19, including 6,475,346 deaths, have been reported to WHO. As of 23 August 2022, a total of 12,449,443,718 vaccine doses have been administered (https://covid19.who.int/). Omicron is a variant of Severe Acute Respiratory Syndrome Coronavirus 2 (SARS-CoV-2) designated as a “Variant of Concern (VOC)” by the World Health Organization (1). In addition, the virus is highly contagious and spreads fast (2). In Shanghai, China, the virus has rocketed exponentially since April 2022 (3). However, whether our vaccines will still protect us after the virus mutates is a concern we all share.

It was reported by a meta-analysis study (4) that the protection rate of vaccine against alpha, beta, gamma, delta were 85, 75, 54, and 74%, respectively. Although the protective effect of different strains varies, vaccines are effective in preventing circulating strains infection. In terms of the newly mutated Omicron, a research showed that the effect of the vaccine in preventing the infection of the Omicron strain exibited a reduction in comparison to the other four new coronavirus variants (5). To date, there is no large sample study to investigate the effect of vaccination on symptoms and length of stay in Omicron patients. To tackle this critical clinical problem, from April 1 to May 30, 2022, the clinical data of COVID-19 patients from the national exhibition and convention center (Shanghai) Fangcang shelter hospital were collected and analyzed.

This study aims to demonstrate the clinical characteristics and the length of hospital stay (LOS) of vaccinated patients from the largest municipal-level Fangcang shelter hospital and to determine the effect of vaccination on Omicron.

## Materials and methods

### Study design

This was a retrospective, single-center, observational cohort study based on patients who were diagnosed with mild or asymptomatic COVID-19 according to classified diagnosis and Treatment Protocol for Novel Coronavirus Pneumonia (9th edition), which was released by the National Health Commission of China (http://www.nhc.gov.cn/yzygj/s7653p/202203/b74ade1ba4494583805a3d2e40093d88.shtml) from the national exhibition and convention center i.e., Fangcang hospital in Shanghai, China. The mild and asymptomatic patients were included in this study since only these two types of patients can be treated in the Fangcang shelter hospitals. Among them, mild cases were defined as patients with mild symptoms (low fever, slight fatigue, dysosmia, dysgustia, etc.) and without abnormalities on chest CT whereas asymptomatic cases were defined as patients with positive reverse transcriptase-polymerase chain reaction (RT-PCR) results and without any clinical symptoms. The diagnosis was performed according to the same protocol. The protocol, case report form, and informed consent form were reviewed by the Ethics Committee of the Second Affiliated Hospital of Army Medical University. The study was initiated after receiving written approval from the Ethics Committee (Approval number: 2022-280-01) and written informed consent of participants in vernacular language. Confidentiality of all the data was maintained.

### Eligibiity

Consenting patients between the ages of 12 and 80, with symptomatic SARS-CoV-2 infection or a positive PCR, were considered eligible. Patients who had received at least one dose of vaccine were placed in the vaccinated group, while those who had not received any vaccine were in the non-vaccinated group. At present, five COVID-19 vaccines have been approved for use in China. According to the technical route, these five vaccines are divided into three categories, including inactivated vaccines, adenovirus vector vaccines and recombinant vaccines. Patients vaccinated with adenovirus vaccine were excluded because the sample size for that cohort was very low. Each admitted patient received one RT-PCR test with the oropharyngeal swab sample every morning during hospitalization. The inpatients would be discharged if they (1) had two consecutive negative RT-PCR findings at least 24 h apart, without any restrictions on the total LOS; (2) had any relieved clinical symptoms.

### Data collection

Demographic data, such as age and gender, were recorded on the case report form. Next, the following data were recorded: date of admission; date of positive RT-PCR test, dates of COVID-19 diagnosis (confirmed by the positive RT-PCR results), date of discharge, disease category (mild or asymptomatic), the medical history including hypertension, diabetes, and other self-reported comorbidities, vaccination. Moreover, the lowest cycle threshold (Ct) values of the RT-PCR tests were documented for both the Ct-ORF and Ct-N. All data were directly obtained from the electronic medical records of the participants.

### Statistical analysis

All analyses were performed using IBM SPSS Statistics, version 26. Continuous variables with normally distributed data were expressed as the means ± standard deviation (SD). Categorical data were expressed as the composition ratio and percentage. The qualitative data were evaluated using Pearson's chi-squared test with continuity correction or the Fisher's exact probability method, while comparisons of quantitative variables between two groups were performed using an independent-sample *t*-test or the Mann-Whitney test, depending on normality of data. A *p*-value < 0.05 was considered statistically significant. The results were expressed as hazard ratios (HR) with associated 95% confidence intervals (CI).

## Results

### Demographics and characteristics of the participants

**Figure 1:**
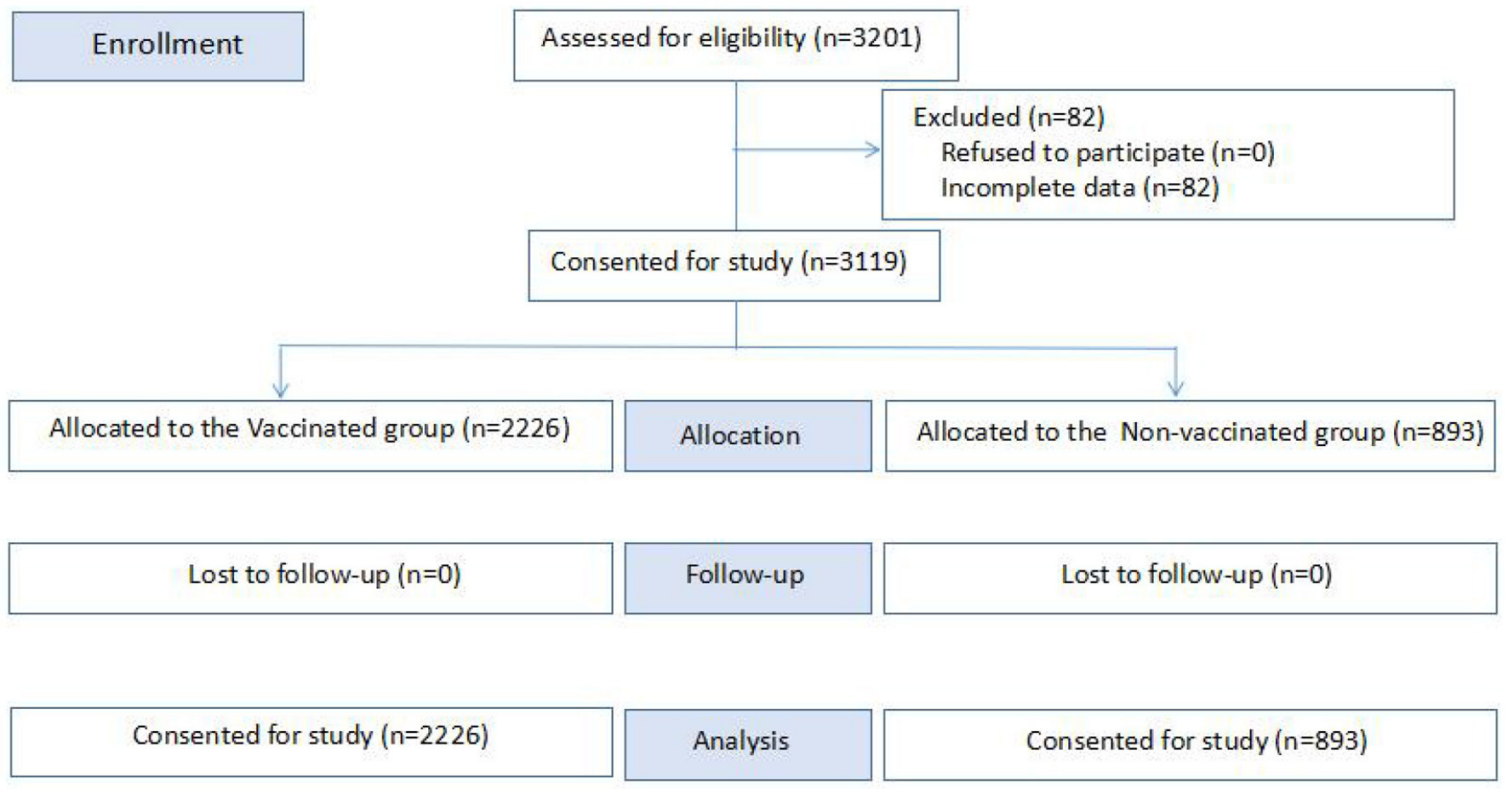
Case selection chart.

Three thousand two hundred one patients were initially enrolled in the study. Of these, 82 (2.6%) patients were excluded due to incomplete case data. Three thousand one hundred nineteen patients were finally included consisting of 2,226 (71.4%) patients in the vaccinated group and 893 (28.6%) patients in the non-vaccinated group before admission (Figure 1). The average LOS of the total study sample was 15.58 ± 2.832 days (range: 4–45 days). There were 1,997 (60.4%) males and 1,122 (35.9%) females, with a mean age of 47.22 ± 15.859 years and a diagnosis of SARS-CoV-2 infection confirmed by RT- PCR *via* an oropharyngeal and nasopharyngeal swab. No significant differences were found between the vaccinated group and the non-vaccinated group regarding the age (*p* = 0.272), the number of ethnic Han patients (*p* = 0.317), or marital status (*p* = 0.390). No chronic comorbidities were confirmed in 2,423 (77.7%) patients, while 696 patients presented one or more comorbidities (hypertension 13.8%, diabetes 5.7%, hypertension with diabetes 1.4%, respiratory diseases 0.3%, liver diseases 0.2%, cardiovascular diseases 1.0%), as shown in Table 1.

**Table 1:** Baseline characteristics of patients with SARS-CoV-2 infection (*n* = 3,119).

	**Enrolled patients**	**Vaccinated group**	**Non-vaccinated group**	***p*-value**
	**(*n* = 3,119)**	**(*n* = 2,226)**	**(*n* = 893)**	
Age, (years)	47.22 ± 15.859	47.01 ± 15.082	47.75 ± 17.646	0.272
Ethnic han patients, (*n* %)	3,054 (97.9%)	2,176 (97.8%)	878 (98.3%)	0.317
Gender				**0.001**
Male, (*n* %)	1,997 (64.0%)	1,470 (66.0%)	527 (59.0%)	
Female, (*n* %)	1,122 (36.0%)	756 (34.0%)	366 (41.0%)	
Marital status, (*n* %)				0.390
Married	2,149 (68.9%)	1,545 (69.4%)	604 (67.6%)	
Unmarried	882 (28.3%)	625 (28.1%)	257 (28.8%)	
Divorced	83 (2.7%)	53 (2.4%)	30 (3.4%)	
Widowed	5 (0.2%)	3 (0.1%)	2 (0.2%)	
**Comorbidity, (*****n*** **%)**
Hypertension	429 (13.8%)	293 (13.2%)	136 (15.2%)	0.130
Diabetes	178 (5.7%)	109 (4.9%)	69 (7.7%)	0.110
Hypertension with diabetes	43 (1.4%)	28 (1.3%)	15 (1.7%)	0.361
Respiratory diseases	9 (0.3%)	5 (0.2%)	4 (0.4%)	0.293
Liver diseases	6 (0.2%)	4 (0.2%)	2 (0.2%)	0.799
Cardiovascular diseases	31 (1.0%)	20 (0.9%)	11 (1.2%)	0.396

IQR, interquartile range; Ct, cycle threshold.

The value in bold represents *P* < 0.05.

### The clinical outcomes between the vaccinated group and non-vaccinated group

The differences between the vaccinated group and non-vaccinated group regarding the LOS, classified diagnosis and outcomes during the hospital stay were noted (Table 2). The average length of stay in the vaccinated group was significantly lower than that in the non-vaccinated group (15.48 ± 2.708 vs. 15.85 ± 3.102, *p* = 0.001). Similarly, vaccination also varies in classified diagnosis (*p* = 0.001). More asymptomatic patients were observed in the vaccinated group than in the non-vaccinated group (70.4 vs. 64.5%, *p* = 0.001).

**Table 2:** In-hospital stays and outcome differences between the two groups (*n* = 3,119).

	**Enrolled patients**	**Vaccinated group**	**Non-vaccinated group**	***p*-value**
	**(*n* = 3,119)**	**(*n* = 2,226)**	**(*n* = 893)**	
LOS	15.58 ± 2.832	15.48 ± 2.708	15.85 ± 3.102	**0.001**
Disease category, (*n* %)				**0.001**
Mild patients	975 (31.3%)	658 (29.6%)	317 (35.5%)	
Asymptomatic patients	2,144 (68.7%)	1,568 (70.4%)	576 (64.5%)	

The value in bold represents *P* < 0.05.

### Subgroup analysis for age group

A subgroup analysis was performed since the age did not differ between the groups (Table 3). We found that the patients in the juvenile group and the young group were vaccinated irrespective of the LOS and the type of symptoms (*p* > 0.01). However, the LOS in the middle-aged (15.40 ± 2.547 vs. 15.84 ± 2.896, *p* = 0.007) and elderly groups (15.67 ± 2.650 vs. 16.20 ± 3.039, *p* = 0.014) was significantly different in the two groups. There were more asymptomatic patients than mild patients in both the middle-aged group and the elderly group whether they were vaccinated or not (*p* < 0.05).

**Table 3:** Subgroup analysis for age group (*n* = 3,119).

**Age group**			**Vaccinated group**	**Non-vaccinated group**	***t*–value or *chi***	***p*-value**
			**(*n* = 2,226)**	**(*n* = 893)**		
Juvenile (12–18)	LOS	15.16 ± 2.964	14.89 ± 2.340	0.508	0.613
	Disease category, (*n* %)	Asymptomatic patients (*n* = 98)	64 (65.3%)	34 (34.7%)	0.896	0.239
		Mild patients (*n* = 22)	12 (54.5%)	10 (45.5%)		
Youth (19–35)	LOS	15.53 ± 3.094	15.59 ± 3.597	−0.208	0.835
	Disease category, (*n* %)	Asymptomatic patients (*n* = 466)	326 (54.5%)	140 (45.5%)	0.003	0.514
		Mild patients (*n* = 205)	143 (69.8%)	62 (30.2%)		
Middle-aged (36–59)	LOS	15.40 ± 2.547	15.84 ± 2.896		**0.007**
	Disease category, (*n* %)	Asymptomatic patients (*n* = 1,054)	837 (79.4%)	217 (20.6%)	10.113	**0.001**
		Mild patients (*n* = 503)	363 (72.2%)	140 (27.8%)		
Elderly (>60)	LOS	15.67 ± 2.650	16.20 ± 3.039	−2.465	**0.014**
	Disease category, (*n* %)	Asymptomatic patients (*n* = 526)	341 (64.8%)	185 (35.2%)	4.208	**0.025**
		Mild patients (*n* = 245)	140 (57.1%)	105 (42.9%)		

LOS, Length of hospital stay.

The value in bold represents *P* < 0.05.

## Discussion

Judging from the current epidemic situation, the prevention and control of the COVID-19 epidemic is repetitive and long-term (6). In the previous waves of the epidemic, strong measures were taken to curb the epidemic spread. Nevertheless, the constant Omicron variant also leaves us with huge medical challenges. To the best of our knowledge, immunity through vaccination is the key to fighting the virus (4). Some researchers (7, 8) reported that the successful development and use of the vaccine had brought new hope for combating the epidemic and vaccination could be effective in reducing the infection, hospitalization and death. Lopez (9) demonstrated that COVID-19-related hospitalizations could be reduced with single-dose vaccination by about 80% and related deaths by 85% among seniors. Furthermore, as one of the countries with the highest vaccination rates, it has witnessed a 25% drop in Malaysia in COVID-19-related hospitalizations and a 22% drop in ICU (intensive care unit) incidence in the short term (10).

So how do vaccines work against Omicron? In our study, 71% of vaccinated patients were still infected by Omicron. This may be due to the fact that the Omicron may escape the immunity of the vaccine and evade the efficacy of the antibody, contributing to the low effectiveness of existing vaccines against the Omicron (11, 12). A South African study showed that Omicron reduced the effectiveness of Delta's vaccine against infection from 80 to 33% (13). Another study in the United States indicated that 5 patients were infected by Omicron within 14 days after vaccination (14). Thus, the vaccine's protection against omicron is greatly reduced, and even those who have previously been infected with other variant virus may still be infected with Omicron (15, 16). The above study in South African (13) may shed light on the fact that serum from 12 people who received the Pfizer-BioNtech vaccine was 40 times less effective against the Omicron than an earlier SARS-CoV-2 strain.

However, our results exibit shorter hospital stays for vaccinated patients than non-vaccinated ones. Taking into consideration all of our included patients were asymptomatic or mild, and there was no evidence of pneumonia on imaging, our discharge criteria were: (1) body temperature returned to normal for more than3 days; (2) improvement of respiratory symptoms; and (3) two consecutive negative nucleic acid tests. It is well known that shorter hospital stay means faster recovery. A previous study demonstrated that partially and fully vaccinated cases were highly protected against hospitalizations related to SARS-CoV-2 Alpha or Delta (17). In terms of the latest variant of the Omicron virus, it was reported that although it reduced the effectiveness of the Pfizer-BioNTech's COVID-19 vaccine, the vaccine could still lower the likelihood of hospital admission (18). Moreover, it has been confirmed that patients infected with omicron recover quicker after vaccination (19, 20).

During the Omicron wave in Shanghai, almost all the asymptomatic or mildly symptomatic patients underwent our standardized treatment. Some studies claim that severe disease can still be prevented by vaccination, because it is unaffected by Omicrons mutation. The surface structure of Omicron spike protein is targeted by T cells, which usually emerge after vaccination (21). Perhaps for this reason, although we do not have critically ill patients, patients who have been vaccinated are more likely to undergo asymptomatic infections. It was reported that a new COVID-19 lineage has been mapped with mutations in the N-terminal and receptor-binding domains of the spike protein that are known targets of neutralizing antibodies (22–24). Omicron contains numerous mutations in the envelope, spike, membrane, nucleocapsid, and non-structural proteins that lead to increased viral transmissibility. A particular cluster of mutations (H655Y, N679K, and P681H) adjacent to the S1/S2 furin cleavage site could facilitate spike protein cleavage, which contributes to enhanced transmissibility (25). However, despite the high prevalence of the COVID-19 variant of concern, the efficacy of the vaccine remains high. Our findings also demonstrated that a COVID-19 vaccine based on the Wuhan-Hu-1 original strain can elicit cross-protective efficacy against the new variant. Non-neutralizing antibodies against COVID-19 variants may be retained because they are not limited to the N-terminal or receptor-binding domains where most mutations occur (26).

Our study documented that both the vaccination or the lack of it had less effect on adolescents and young adults, but a larger impact on middle and old individuals. As the elderly grow older, the body's immune system function declines, and most elderly have underlying diseases such as diabetes, hypertension, and potential malnutrition, which makes the elderly population more susceptible to be infected with the new coronavirus and suffer from more severe diseases after infection than other age groups (9, 27). It is universally acknowledged that older people have weaker physical functions than people of other ages. Thus, the risk of moderate to severe disease for the aged is much higher than that of adolescents and children once infected with COVID-19, which may aggravate the original underlying diseases of the elderly (28). Hit by multiple symptoms, older citizens may have more severe symptoms than other age groups, and LOS are correspondingly longer. Therefore, in our research, the vaccine is more protective for the elderly than other age groups, which is consistent with a previous survey (29), which showed that people aged 65–74 years had a 5-fold enhanced possibility of hospitalization after infection with novel coronavirus compared with people aged 18–29 years.

### Limitations

Our study has the following limitations. First, this is a retrospective study with unavoidable bias. Second, there is a lack of medium and long-term follow-up of the included patients. In addition, this center is only for asymptomatic and mild patients, and the results obtained are difficult to extrapolate the correlation between vaccination and asymptomatic carriers comprehensively.

## Conclusions

In this retrospective, observational cohort study, COVID-19vaccine has proven to be effective against the mutated novel coronavirus, which can obviously reduce LOS in the Fangcang shelter hospital during the wave of Omicron epidemic in China. Patients vaccinated with COVID-19 vaccine are more likely to be asymptomatic, and the effectiveness is more evident for elderly patients than other age groups.

## Data availability statement

The raw data supporting the conclusions of this article will be made available by the authors, without undue reservation.

## Ethics statement

The studies involving human participants were reviewed and approved by the Ethics Committee of the Second Affiliated Hospital of Army Medical University. Written informed consent to participate in this study was provided by the participants' legal guardian/next of kin.

## Author contributions

Study design, review, and editing: LC, XZ, and SC. Conceptualization: CY and ZF. Data collection: JQ, SX, and WX. Statistics: WH, LZ, LY, GY, and SR. Software and formal analysis: CY, JQ, SX, and WX. All authors have read and agreed on the published version of the manuscript.

## Funding

This study was funded by the Excellent Talent Pool of Army Military Medical University (Number: 2019R014).

## Conflict of interest

The authors declare that the research was conducted in the absence of any commercial or financial relationships that could be construed as a potential conflict of interest.

## Publisher's note

All claims expressed in this article are solely those of the authors and do not necessarily represent those of their affiliated organizations, or those of the publisher, the editors and the reviewers. Any product that may be evaluated in this article, or claim that may be made by its manufacturer, is not guaranteed or endorsed by the publisher.
